# Time trends in preemptive kidney transplantation in Europe: ERA Registry Figure of the month

**DOI:** 10.1093/ckj/sfaf304

**Published:** 2025-10-10

**Authors:** Vianda S Stel, Alberto Ortiz, Anneke Kramer

**Affiliations:** ERA Registry, Department of Medical Informatics, Amsterdam UMC–Location University of Amsterdam, Amsterdam, the Netherlands; Amsterdam Public Health Research Institute, Quality of Care, Amsterdam, the Netherlands; Department of Nephrology and Hypertension, IIS-Fundacion Jimenez Diaz UAM, Madrid, Spain; Department of Medicine, Universidad Autonoma de Madrid, Madrid, Spain; ERA Registry, Department of Medical Informatics, Amsterdam UMC–Location University of Amsterdam, Amsterdam, the Netherlands; Amsterdam Public Health Research Institute, Quality of Care, Amsterdam, the Netherlands

**Figure 1:**
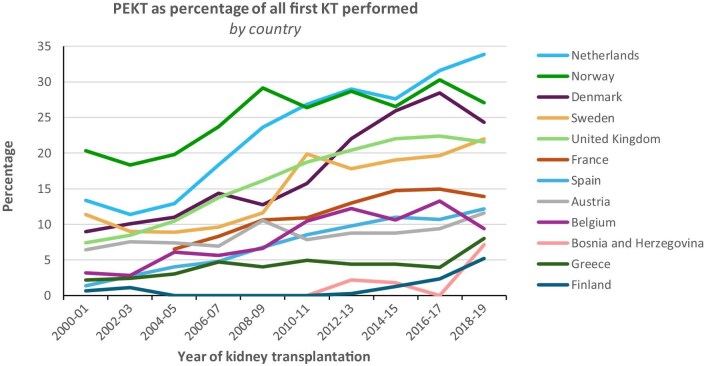
Preemptive kidney transplantation as percentage of all first kidney transplantations performed, by country. **Explanation:** In most countries, the proportion of patients receiving a first kidney transplant while not yet receiving dialysis has increased from 2000 to 2019. However, in 2018–19 there were striking differences between countries varying from 5.2% in Finland to 33.9% in the Netherlands. Notably, Finland—after recognizing that their preemptive transplant rate was the lowest in Europe—implemented targeted interventions that successfully improved their rates. This example highlights how renal registries can play a vital role in identifying or generating hypotheses about underlying clinical issues, ultimately contributing to the optimization of kidney care.

**Source:** Kramer et al. NDT 2024, https://doi.org/10.1093/ndt/gfae105, Figure 2A.

